# *In-situ* gel drug delivery system as a viable approach to periodontal therapy: A comprehensive review

**DOI:** 10.34172/japid.025.3827

**Published:** 2025-10-02

**Authors:** Supreeth Sirangala Vivek, Venkatesh Dinnekere Puttegowda, Joysa Ruby Joseph Rajarathinam, Gunashekar Dhanapal, Naveen Kumar Karimaranahalli Banappa, Chaithanya Kadalu Mahendra, Pooja Rangadham

**Affiliations:** Department of Pharmaceutics, Acharya & BM Reddy College of Pharmacy Soldevanahalli, Hesaraghatta Road, Bengaluru-560107 Karnataka, India

**Keywords:** Antibiotics, Carbopol, Controlled drug delivery, In situ gel, Periodontal disease

## Abstract

This narrative review summarizes recent advances in in situ gel drug delivery systems for periodontal therapy, focusing on formulation strategies, the pharmaceutical agents involved, and their clinical applications. A comprehensive search was conducted on PubMed, Scopus, and Google Scholar for English-language articles published from 2000 to 2024 using relevant keywords. Articles were selected based on their clinical significance, scientific rigor, and relevance to periodontal treatment. In situ gels use physiological stimuli such as pH, temperature, or ions for sol-to-gel transition, enabling sustained and localized drug release into periodontal pockets. These systems aim to improve therapeutic efficacy and reduce systemic side effects. However, limitations like inconsistent gelation, low mechanical strength, and limited long-term clinical data hinder widespread clinical adoption. Future research should focus on formulation optimization and rigorous clinical trials to facilitate the integration of in-situ gels into routine periodontal care.

## Introduction

 Periodontitis is a complex, chronic, inflammatory disease caused by biofilms. Multiple factors contribute to the damage of the periodontal apparatus. Understanding the role of bacteria in periodontal disease is crucial for prevention and treatment.^1^ Loss of tissue attachment, loss of alveolar bone, and formation of pockets are the primary signs of periodontal disease symptoms. Clinical studies have shown that scaling and root planing, along with the removal of bacterial deposits from the tooth surface, effectively reduce probing pocket depths. Antimicrobial strategies have been developed and tested to eliminate all microorganisms associated with periodontal disease since mechanical debridement alone is insufficient for this purpose.^2^ Hundreds of different types of anaerobic and aerobic bacteria make up the oral microbiota. Deep-layer microorganisms adhere to and are compactly arranged against the tooth, while more motile forms inhabit the superficial layers. Biofilms refer to the growth on tooth surfaces in the form of complex mixed colonies that exist interdependently.^3^ The most common microorganisms causing periodontal disease are Gram-negative anaerobic bacilli, including many anaerobic spirochetes and some anaerobic cocci. *Porphyromonas gingivalis, Bacteroides forsythus, Prevotella intermedia, Actinobacillus actinomycetemcomitans, *and *Treponema denticola* are the main microorganisms associated with severe periodontal lesions.^4^ The goals of modern periodontitis treatment are to reduce infection, decrease inflammation, and establish a clinical state consistent with periodontal health. The first line of treatment for periodontitis is usually non-surgical, encompassing scaling and root planing (SRP) alongside oral hygiene instructions.^5^ In the treatment of periodontitis, systemic antibiotics should not be used alone. Systemic antibiotics are effective antibacterial agents for treating periodontal diseases when used in conjunction with adequate mechanical debridement to disrupt subgingival biofilms. The best antibiotic, dosage, duration, and timing are all subjects of debate. In cases where aggressive periodontitis is present, adjunctive systemic antibiotics should be considered. Although data indicate that supplemental systemic antibiotics provide additional therapeutic benefits in treating persistent periodontitis in deep pockets, the decision to prescribe antibiotics should be made on an individual basis.^6,7^ The rise in antibiotic resistance began to be acknowledged in the 1980s. The medical profession has overlooked the fact that microorganisms have been using antibiotics to combat each other for over two billion years, and during these decades of arrogance, they have developed resistance mechanisms to overcome them. Antibiotics will never fully “defeat” bacteria. The resistance is unavoidable.^8^ Non-steroidal anti-inflammatory drugs (NSAIDs) are among the most commonly used medications worldwide. They are prescribed to treat fever, inflammation, and pain. The term “NSAID” can apply to a broad category of medications used to manage chronic rheumatic conditions related to periodontitis.^9,10^

 An in situ gel system with a local anesthetic is preferable to an emulsion-based drug delivery system, as it is easy to apply to the periodontal pocket, remains at the application site for the required duration, provides a quick onset of anesthesia that lasts throughout the dental procedure, and can be easily rinsed out with water afterward. Topical formulations for local anesthetics in periodontal pockets are rare, but in situ gel systems have garnered significant interest from pharmaceutical developers as a drug delivery vehicle for dermal, nasal, ocular, oral, buccal, vaginal, rectal, and parenteral administration.^11^ The advantages of in situ polymer delivery systems include ease of drug delivery, decreased administration rates, controlled release, improved patient tolerance, and convenience; they are also biocompatible and biodegradable, fostering interest in these formulations.^12^ Additionally, the formulation is simpler, which lowers manufacturing costs and investments.^13^ There are three types of in situ gelling systems: ion-activated systems like gellan gum and sodium alginate; temperature-dependent systems such as Pluronic and polymethacrylates; and pH-triggered systems like carbopol and cellulose acetate phthalate.^14,15^ This local medication therapy raises the question of whether to use it as an adjunctive technique to scaling and root planing or as a definitive treatment alternative. There are several reasons to avoid using local medication delivery systems as an alternative technique: Patients with adult periodontitis treated by scaling and/or root planing achieve equivalent clinical outcomes compared to those who received drug therapy alone, which also avoids introducing the risk of drug-resistant bacterial strains; root planing can help disrupt and eliminate biofilms that are less likely to respond to local drug delivery; root surface instrumentation can remove calculus, which may contain endotoxins that inhibit plaque accumulation; and scaling and root planing in maintenance patients may be less costly when applied to all affected teeth.^16^ Periodontal treatment often employs locally applied antibiotics like tetracycline, doxycycline, metronidazole, and minocycline due to their antimicrobial action against both Gram-positive and Gram-negative organisms. As tetracycline analogs, doxycycline and minocycline have enhanced lipid solubility and longer action, while metronidazole effectively combats anaerobic infections. Table 1 presents the critical features of these antibiotics.^1^

## Methods

 An extensive search of PubMed, Scopus, and Google Scholar was performed until March 2024, with the keywords “in situ gel” or “gel-forming system” in combination with “periodontal disease” or “periodontitis” or “periodontal therapy” with “drug delivery” or “mucoadhesive” or “local delivery.” A manual search from the bibliographies of the selected articles and relevant reviews was also performed.

 The areas of interest for the review included:

1. Which in situ gel-based drug delivery systems have been developed for periodontal therapy?
2. On what formulation bases, mechanisms of action, and clinical effectiveness have they been reported?

Articles eligible for inclusion discussed in situ gel systems for localized drug delivery, specifically in periodontal treatment. Only articles in English with full-text availability were included. Exclusion criteria included studies involving routes other than periodontal (such as ophthalmic, nasal, etc.), gels not classified as drugs, animal or in vitro studies with no clinical correlation, abstracts without full data, as well as patent applications and editorials without experimental proof.

 Titles and abstracts were screened first. Full texts were assessed if they met the inclusion criteria or if their abstracts were considered unclear. Both authors independently assessed all studies that were finally included. The extracted data consisted of formulation compositions, type of gel system (thermoresponsive, ion-sensitive, pH-sensitive), active pharmaceutical ingredients, polymer characteristics, route of administration, drug release profile, and any clinical findings.

## Results

 A total of 1,126 articles were retrieved from PubMed, Scopus, and Google Scholar. The titles, abstracts, and full texts were screened against the inclusion and exclusion criteria, leading to a final total of 42 articles selected for the review. The selected studies were segregated and systematically discussed under six themes: thermoresponsive in situ gels, ion-sensitive gels, pH-responsive systems, mucoadhesive and polymer-based formulations, drugs commonly incorporated in periodontal gels, and clinical evaluation of their therapeutic effectiveness. These categories greatly helped in organizing the findings related to formulation design, gelation triggers, drug release profile, and periodontal outcome systematically.

###  Periodontal disease

 Periodontal disease is a chronic inflammatory condition characterized by the formation of periodontal pockets, gingival inflammation, loss of connective tissue attachment, and eventual tooth loss.^17,18^ The traditional definition of periodontal disease refers to the progressive degeneration of the hard and soft tissues of the periodontal complex, caused by dysbiotic bacterial colonies coexisting symbiotically with aberrant immune responses in the gingiva and periodontal tissues.^19^

**Figure 1:**
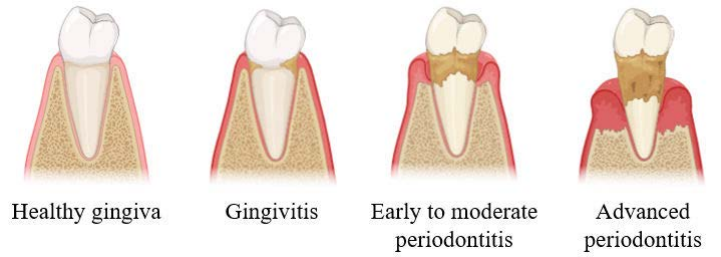
Progression of periodontal disease: The stages of periodontal disease through which clinically infected periodontal tissue goes, starting with a healthy gingiva, progressing through a stage of gingivitis, and ending with periodontitis^3^

 The progression of periodontal disease from healthy gingiva to advanced periodontitis is characterized by increasing pocket depths, alveolar bone loss, and inflammation (Figure 1).

####  Etiopathogenesis of periodontal diseases

 Since Anton von Leeuwenhoek’s report of the presence of “animalcules” in dental plaque, it has been understood that these bacteria are present in the oral cavity. For over a century, the bacterial etiology of periodontal disorders has been studied and developed with technological advancements in characterization and identification.^20^

 The etiopathogenesis of periodontal diseases involves a complex relationship between microbial populations. Early colonizers such as *Gemella, Atopobium, Fusobacterium nucleatum, Streptococcus sanguis, S. mitis, and Capnocytophaga* species inhabit healthy periodontal tissues, most beneficial to the host.^21,22^ Gingivitis occurs with both Gram-negative (*Campylobacter gracilis, F. nucleatum, Prevotella intermedia, *and*Veillonella*) and Gram-positive (*Streptococcus spp., Actinomyces viscosus, *and*Peptostreptococcus micros*) species, the former being particularly characteristic in pregnancy-related gingivitis.^22-24^ Chronic periodontitis, however, is marked by increased levels of pathogens like *Tannerella forsythia, Campylobacter rectus, P. intermedia, Porphyromonas gingivalis, *and* F. nucleatum*.^22-25^ Localized aggressive periodontitis (LAP) is the focus of *Actinobacillus actinomycetemcomitans, *with*Eikenella corrodens, P. gingivalis, *and* C. rectus*.^26,27^ The microbiological picture of generalized aggressive periodontitis is similar to that of chronic periodontitis because of similar etiologies.^28^ Necrotizing ulcerative gingivitis is most commonly linked with *spirochetes *and *fusobacteria*, such as the newly discovered *Treponema putidum*.^29^ Periodontal abscesses include the presence of bacteria such as *T. forsythia, C. rectus, P. gingivalis, F. nucleatum, P. intermedia, and P. micros*.^30^

####  Classification

 To facilitate more accurate diagnosis and treatment of periodontal and peri-implant diseases, the 2017 American Academy of Periodontology (AAP/EFP) and European Federation of Periodontology classification incorporated staging, grading, and more categories. Practitioners can use this new paradigm to assist in diagnosis, treatment planning, and long-term follow-up. Table 2 outlines the classification.^31,32^

####  Periodontitis clinical signs

 Gingival inflammation, pocket formation, bone loss, clinical attachment loss, tooth mobility, and foul breath, all of which are recognized symptoms of periodontitis. Some of these symptoms include gingival redness, swelling, and tenderness; gingival bleeding; receding gingiva; chronic bad breath; pain or discomfort; changes in occlusion; and pus formation between the teeth and gingiva. As periodontitis progresses, increased tooth mobility, root surface exposure, and the formation of periodontal abscesses may occur. Other symptoms consist of bleeding gums, gingival recession, chronic bad breath, soreness or discomfort, changes in occlusal alignment, and pus formation between the gums and teeth.^33,34^

####  Risk factors for periodontal disease

 A vast array of risk factors contribute to the development of periodontal disease. Over half of the instances are brought about by smoking, a factor also linked to cancer and oral ulcers.^35,36^ While more studies are required to fully appreciate the contribution of nutrition, poor diet and vitamin C deficiency can exacerbate periodontal diseases.^37^ Beyond its contribution to advanced periodontitis, excessive consumption of alcohol is often concomitant with smoking and poor diet.^38,39^ Through physiological mechanisms, stress is involved in susceptibility to periodontal disease.^40,41^Additionally, genetics is also involved, as certain inherited characteristics affect dental and gingival health.^42,43^

####  Treatment

 Medications are used to enhance the results and encourage recovery from periodontitis. There are regenerative agents such as bone morphogenetic protein-7 (BMP-7) and zinc-hydroxyapatite nanoparticles, and antibiotics such as levofloxacin, minocycline, tetracycline, ornidazole, metronidazole, and doxycycline that are effective in treating periodontal disease.^44^ Nonsteroidal anti-inflammatory drugs used to treat periodontal diseases are aspirin, flurbiprofen, ibuprofen, naproxen, piroxicam, diclofenac sodium, meloxicam, nimesulide, etodolac, and celecoxib.^45^For periodontal treatment, several local sustained drug delivery systems have been developed; these can be broadly classified generically as reservoir and matrix types. Depending on the use, these are made up of fibers, films, injectables, microcapsules, and gels/ointments, which incorporate both biodegradable and non-biodegradable components. Table 3 summarizes the categories and materials used in these systems.^46^

###  In situ gel systems

 In situ gel administration is a good method of treating periodontitis because it continuously and carefully administers medication to the infected location. Researchers used ingredients such as zein, borneol, piperine, and curcumin to develop a formulation that targeted the inflammatory state linked to dysbiosis in periodontitis, thus showing antibacterial properties.^34^ To deliver these gels in a liquid state that turns into a gel when it comes into contact with the target site, so that there can be sustained drug release and better therapeutic results.^34-47^

 In situ gels comprise hydroxy propyl methyl cellulose (HPMC), sodium alginate, and xanthan gum; these polymers improve the viscosity and control the rates of drug release. These systems have been used to produce in situ gel for administration routes such as ocular, oral, and nasal for the treatment of ulcer diseases, eye infections, rheumatoid arthritis, and so on.^48^

**Figure 2:**
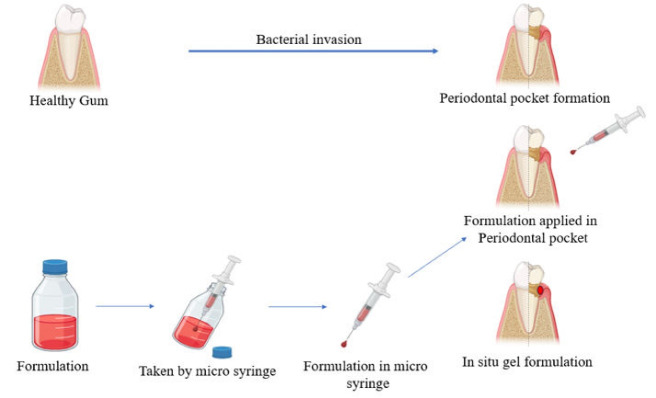
Administration of in situ gel: the formulation is inserted directly into the periodontal pocket and undergoes a sol-to-gel transition at the site^14^

 Using in situ gel involves administering the formulation in a microsyringe and directly introducing it into the periodontal pocket, where it undergoes sol-to-gel transition at the infected site (Figure 2).

###  Formulation

####  Selection of a vehicle

 The right vehicle must be selected before the in situ gelling system can be prepared. Polymer dispersions were thus prepared in several buffer solutions, among which were phosphate-buffered IP (pH = 6.0), citrophosphate-buffered IP (pH = 5.0, 6.0), and acetate-buffered IP (pH = 4.0, 5.0).^49^

####  pH-induced system

 All the sensitive polymers to pH contain either basic or acidic groups, which, depending on the pH of their surroundings, release or accept a proton. Gel swelling is directly proportional to the increase in external pH when anionic groups are present; otherwise, it decreases when cationic groups are present in the polymer. It comprises in situ gel from a variety of pH-sensitive polymers, such as carbopol; the physiological shift in pH has caused the solution-gel transition.^50^

####  In situ gel systems activated by temperature

 Before they harden inside the targeted tissue, organ, or body cavity, these injectable solutions can be given minimally invasively. A temperature-activated solution is used in mucoadhesive formulations to gel transition polymer over a temperature range of 25–37 °C. Polymers with a low critical solution temperature of roughly 32 °C undergo a phase change at body temperature. Poloxamer is one of the numerous thermosensitive polymers that can be applied to in situ gel systems.^51^

####  The ion-activated mechanism

 Gelation is triggered by a change in the ionic strength of the implanted solution. The osmotic gradient surrounding the gel surface regulates the rate of gelation. Gelation is triggered by a change in the implanted fluid’s ionic strength. The pace of gelation is regulated by the osmotic gradient surrounding the gel surface. Electrolytes such as Ca^2 +^, Mg^2 +^, and Na ^+^ cations, which are present in fluids available in the oral cavity, are crucial in the initiation of gelling when body cavities are used to deliver solutions. Alginates, hyaluronic acid, gellan gum, and gelrite are a few examples of polymers.^52^

####  Preparation of in situ gel

 In situ gel is prepared by the cold method. A measured amount of polymer is slowly added to water inside a beaker with constant stirring using a magnetic stirrer. Throughout the process, the temperature of the water is maintained at 4 °C. This solution is refrigerated overnight. The preservative is dissolved in hot water to prepare the solution. After cooling, it is mixed with the above dispersion. The weighed amount of medication is dissolved in the solvents. The medication solution is mixed with the polymer dispersion as described earlier.^53^

###  Characterization

 The following parameters are used to analyze and characterize the in situ gel:

####  Clarity

 A white and black background could be used to visually review the solution and assess its clarity.^54,55^

####  Texture analysis

 Using a texture profile analyzer, the gel’s consistency, stiffness, and cohesiveness are measured in situ. This mainly shows the strength of the gel and the ease of its administration in vivo; a higher adhesiveness rating is necessary to maintain close contact with the mucus surface.^56^

####  pH of gel

 The pH can be found by placing the formulation in a beaker, adding 1 mL of NaOH drop by drop at a time, and constantly stirring. To check pH, a pH meter is used.^57^

###  Rheological studies

 Cone and plate viscometers and Brookfield viscometers are used for viscosity. The sample tube of the in situ gel composition is filled. The viscosity of the formulation should be between 5 and 1000 mPas before gelling, and it will be between 5050,000 and 500,000 mPas (millipascal-second) once the periodontal pocket activates the ion gel.^58^

###  Swelling studies

 A cell, which has been fitted with a thermal jacket to hold the temperature at a relatively constant level, is used for swelling investigations.^59^ Synthetic gingival crevicular fluid^54^ is available within the cell. One milliliter of the conditioned solution is placed in the dialysis bag, and the latter is submerged in the swelling medium. The latter is equilibrated at 370ºC. At predetermined time intervals, the bag is removed from the medium and its weight is measured. The swelling of the polymer gel as a function of time is computed and measured using the following relationship.^60^

 %St = 100/W0 (Wt - Wo)

 where St represents the swelling at the time “t,” Wo is the initial weight of the gel, and Wt is the gel’s final weight.

###  High-performance liquid chromatography

 Reversed-phase mode is applied with the HPLC system. For analysis, a C18 Nova pack, a packed column of length (150 × 3.9 mm id), is used.^61^

###  Fourier transformer infra-red

 FTIR studies explore whether drugs interact with excipients or not. KBR pellets are employed to register FTIR graphs of both the pure drugs and combinations of drugs with excipients.^62^

###  In vitro drug release studies

 An in vitro release investigation of an in situ gel solution is conducted using the Franz diffusion cell. The formulation is put in the donor compartment, and freshly made gingival crevicular fluid is placed in the receptor compartment. The donor and receptor compartments are separated by the dialysis membrane, which has a hole size of 0.22 μm. A magnetic stirrer with a thermostat is used to set up the entire device. The temperature of the medium is kept at 37 ± 0.5 °C. Then, 1 mL of the sample that is removed at predetermined intervals of one hour is replaced every 6 hours with a sample volume of fresh medium. The extracted sample is diluted to 10 mL in a volumetric flask with the proper solvent using a reagent blank, and then it is analyzed by a UV spectrophotometer at the proper wavelength. An equation based on the standard calibration curve is used to determine the drug content. The cumulative drug release (CDR) percentage is calculated. For drug release data, curve fitting is performed on the gathered data. The kinetics of Fickian and Korsmeyer-Peppas diffusion mechanisms are examined using the best-fit model.^54^

###  Antimicrobial activity

 Antimicrobial effectiveness experiments are performed to determine the biological activity of the sol-gel system against bacteria. This is identified using the “cup plate technique” in an agar diffusion medium. The bacterial microbial growth is tested by serial dilution of the microbiological assay compared with known concentrations of standard antibiotic preparation.^54^

###  Accelerated stability studies

 According to the International Conference of Harmonization State Guidelines, the preparation is filled into amber-colored vials and closed with aluminum foil for the short-term accelerated stability study at 40 ± 20°C and 75 ± 5% RH. The sample is checked monthly for in vitro dissolution, rheological evaluation, drug content, clarity, pH, and gelling capacity.^63^

###  Commonly used polymers in the formulation of in situ gel

 In situ gels are increasingly becoming renowned for their therapeutic potential in periodontal disease because they can release medication locally to the site of action in a controlled manner. Various polymers are used to form in situ gels, each of which contributes its characteristics to enhance drug delivery and therapy. The most commonly used polymers for preparing in situ gels for periodontal disease treatment are temperature-sensitive poloxamer 407, ion-sensitive gellan gum, and pH-sensitive carbopol 934P. These polymers enhance the drug’s local action and retention.^64^

 Various polymers, including chitosan, Poloxamer 407, and sodium alginate, are used to formulate in situ gels for periodontal therapy due to their different functions, ranging from controlled drug release, mucoadhesion, and biocompatibility. Each polymer possesses its distinct advantages and disadvantages, and this could influence the effective performance of the formulations (Table 4).

###  Limitations of in situ gel systems

 Despite its advantages, in situ gel systems have several limitations, such as insufficient mechanical strength, poor drug loading, poor gelation, and short residence times at the site of application. Precipitation of drug, sensitivity to the environment, limited release control, and stability concerns are additional challenges. To resolve these issues, strategies such as using bio-adhesive polymers, advanced encapsulation technology, and optimization of polymer formulations have been proposed. Table 5 is an overview of these limitations and potential remedies.

###  Efficacy of antibiotics and NSAIDs

 A study thoroughly investigated the efficacy of NSAIDs and antibiotics in the treatment of periodontal disease, with significant advantages shown when combined with SRP or as part of cutting-edge delivery systems, such as in situ gels.

###  Antibiotics in periodontal therapy

*Role*: Antibiotics reduce the microbial load within periodontal pockets by specifically targeting bacteria involved in periodontal diseases, like*Porphyromonas gingivalis *and* Aggregatibacter actinomycetemcomitans*.^4^ Some of the antibiotics include azithromycin, doxycycline, and ornidazole.^7^

*Clinical evidence*: Systemic antibiotics and scaling and root planing significantly reduced probing pocket depths and the clinical attachment loss in comparison to the latter alone, based on a report by Herrera et al.^78^ Local delivery methods, including gels releasing chlorhexidine or doxycycline elevate medication levels in periodontal pockets without many systemic adverse effects.^79^

*Effectiveness*: Isolated application of antibiotics will increase clinical attachment loss and lower probing pocket depths while reducing microbial burden and inflammation.^80^

###  NSAIDs in periodontal therapy

 They are also known as non-steroidal anti-inflammatory medicines.

*Role*: They work by inhibiting the cyclooxygenase enzymes COX-1 and COX-2, thereby reducing prostaglandin synthesis and modulating periodontal inflammation. Naproxen, ibuprofen, and diclofenac are just a few of these.^15^

*Clinical evidence*: Tonetti et al. demonstrated that systemic NSAIDs, including flurbiprofen, resulted in reduced alveolar bone loss during six months in patients with long-term periodontitis.^81^ Administration of NSAIDs via biodegradable gels locally decreased gastrointestinal side effects without causing tissue damage due to inflammation.^82^

*Effectiveness*: There is a significant reduction in the effects of NSAIDs on tissue edema, gingival inflammation, and matrix metalloproteinase-8 (MMP-8) and receptor activator of nuclear factor-kappa-B ligand (RANKL), which are indicators of bone resorption.^83^

###  Combination therapy

*Synergistic benefits*: By targeting both inflammatory pathways and microbiological etiology, NSAIDs combined with antibiotics have demonstrated cumulative benefits. Studies have shown that when treatment is compared as a combination rather than as monotherapy, a greater reduction in PPD and better gingival health status are observed.^84^

*Clinical applications*: Combining antibiotics and NSAIDs in local in situ gel systems allows for sustained release, resulting in increased absorption and decreased systemic side effects.^85^

###  Clinical evidence supporting in situ gel therapy in periodontitis

 Increasing numbers of clinical trials have evaluated the efficacy of in situ gel systems for topical periodontal treatment in recent years. The devices are engineered to release antibacterial or anti-inflammatory agents directly into periodontal pockets for a protracted period to enhance clinical outcomes and patient compliance.

 An in situ gel incorporating ornidazole-loaded microspheres was used in patients with chronic periodontitis in a separate clinical study. Enhanced microbiological and clinical outcomes were achieved through the great rheological properties of the formulation and long-term release of antibacterial activity.^48^

 Based on the review, the application of locally applied antimicrobials, including doxycycline and chlorhexidine gels, as well as scaling and root planing, resulted in fewer systemic side effects and a slight but statistically significant benefit regarding clinical attachment gain and pocket depth reduction.^78^

 When formulated as a gel and used locally, minocycline HCl microspheres significantly reduced the levels of *Porphyromonas gingivalis, Tannerella forsythia*, and other red-complex bacteria. Compared to SRP alone, clinical parameters like clinical attachment level (CAL) and probing pocket depth (PPD) exhibited statistically significant improvements.^79^

 Several in vivo studies have demonstrated that many in situ gels can help improve probing pocket depth and clinical attachment level, as well as enhance antimicrobial capacity for periodontal interventions. This additional evidence suggests the use of drug gels, such as those containing minocycline, chlorhexidine, doxycycline, and ornidazole, as an adjunct to more conventional practice (Table 6).^48,78,79^

**Table 1:** An overview of locally delivered antibiotics used in periodontitis patients

**Locally delivered antibiotic types**	**Description**
Tetracycline	Drugs within a wide range of bacteriostatic activity that act against both Gram-negative and Gram-positive bacteria.
Doxycycline	A long-acting second-generation tetracycline antibiotic.
Metronidazole	This chemotherapy drug is effective against both Gram-positive and Gram-negative anaerobic bacteria.
Minocycline	The most lipid-soluble and potent semi-synthetic tetracycline antibiotic acts on Gram-negative and Gram-positive bacteria, including those with and without cell walls.

**Table 2:** Classification of periodontal disease

**Category**	**Subcategories**
1. Periodontal health & gingival diseases	- Clinical health on intact periodontium - Clinical health of the reduced periodontium - Gingivitis: Biofilm-induced or non-biofilm-induced
2. Periodontitis	- Unified diagnosis: Periodontitis - Staging (I–IV): Based on severity and complexity - Grading (A–C): Based on risk factors and progression rate
3. Necrotizing periodontal diseases	- Necrotizing gingivitis - Necrotizing periodontitis - Necrotizing stomatitis
4. Periodontitis as a manifestation of systemic diseases	- Associated with genetic disorders or systemic diseases (Papillon–Lefèvre syndrome, hematologic disorders)
5. Other conditions affecting the periodontium	- Systemic diseases affecting periodontal tissues - Mucogingival deformities - Occlusal trauma - Tooth/prosthesis-related factors
6. Peri-Implant diseases and conditions	- Peri-implant health - Peri-implant mucositis - Peri-implantitis - Tissue deficiencies around implants

**Table 3:** Systems for local sustained delivery (LSDS )

** Type**	** Sub-Type**	** Material**
1] Reservoir type	Fiber	Non-biodegradable
2] Matrix type	Films	Bio-degradable
		Non-biodegradable
	Injectable	Thermosensitive polymers
	Microcapsule	Gelatin, Chitosan microspheres
	Fiber	Bio-degradable
		Non-biodegradable
	Gel/Ointment	Carbopol, Chitosan, Poloxamer

**Table 4:** Polymers used in the formulation of in situ gels

**Polymer**	**Action**	**Merits**	**Demerits**
Chitosan^34-65^	Mucoadhesive and antibacterial; forms a gel in response to pH	Biocompatible, promotes wound healing, and has good mucoadhesion	pH-sensitive gelation may vary in different oral environments
Pluronic F127^66,67^	Thermoresponsive gelation at body temperature	Easy administration, suitable for sustained drug release	Weak mechanical strength, rapid dissolution in saliva
Poloxamer 407^66-68^	Thermosensitive polymer; gels at physiological temperature	Reversible gelation, biocompatible, suitable for injectable formulations	Low mucoadhesion alone often needs a combination with mucoadhesive polymers
Sodium Alginate^68^	Forms gel in the presence of calcium ions (ion-sensitive)	Biocompatible, non-toxic, forms stable gels	Limited mechanical strength, brittle in dry conditions
Carbopol 934P^65-69^	Swells in water to form a gel; used for viscosity modulation	Good control over drug release enhances adhesion	Can irritate if used in high concentration
Hydroxypropyl Methylcellulose (HPMC)^65-70^	Swells upon hydration, forms a viscous gel	Sustained drug release improves viscosity	Limited responsiveness to external stimuli (e.g., temperature or pH)
Gellan Gum^70^	Ion-sensitive polymer, gels in the presence of cations	Good mucoadhesive properties, clear gel formulation	Requires precise ionic balance for proper gelation

**Table 5:** Limitations of *in-situ* gel systems

**Limitations**	**Methods to Overcome**
Low mechanical strength^71^	Incorporate bio-adhesive polymers (e.g., chitosan, Carbopol) to improve strength and retention.
Poor drug loading efficiency^72^	Use advanced encapsulation techniques like liposomes, nanoparticles, or micelles.
Inconsistent gelation time^73^	Optimize polymer concentration and crosslinking agents for consistent gel formation.
Short retention time at application site^74^	Employ mucoadhesive agents to prolong contact duration.
Limited drug release control^75^	Implement dual polymer systems or controlled-release microcarriers.
Environmental sensitivity (pH/temperature variations)^76^	Develop stimuli-responsive gels with optimized transition points.
Stability issues^77^	Use stabilizers or antioxidants to improve shelf life.

**Table 6:** Summary of clinical evidence

**Study**	**Drug/Formulation**	**Design**	**Findings**
Diasa et al, 2016.^48^	Ornidazole microsphere gel	Clinical evaluation	Sustained drug release; improved antimicrobial profile
Herrera et al, 2002.^78^	Chlorhexidine/doxycycline gels	Systematic review	Adjunctive benefit with scaling and root planing; improved clinical attachment level (CAL) and probing pocket depth (PPD)
Goodson et al, 2007.^79^	Minocycline gel	Randomized controlled trial	Reduced red-complex bacteria; improved probing pocket depth and clinical attachment level

###  Future perspectives

 To improve the efficacy and delivery of NSAIDs and antibiotics in periodontal therapy, researchers have been working to develop innovative technologies, such as nanocarriers, liposomes, and biopolymer-based in situ gels.^86,87^

###  Research gap

 Seemingly great from their promise to locally deliver therapeutic agents to periodontal pockets, significant gaps exist in long-term clinical validation, formulation standardization, and real-world applicability. Bridging these gaps through high-quality clinical trials and patient-centered studies is crucial for enabling their successful implementation in the clinic.

## Discussion

 In situ gel systems represent a promising innovation in periodontal therapy that allows for localized and sustained delivery of the drug directly into periodontal pockets. Compared to systemic treatments, these therapeutic gels enhance the therapeutic efficacy at the site of action while minimizing the systemic side effects, making them promising candidates to treat localized disease states like periodontitis. In situ gel sol-gel transition can be triggered by physiological stimuli such as pH, temperature, or ions, and hence create conditions for better retention and longer contact of the drug. Among the polymers contributing to gelation, drug release, and mucoadhesion are Poloxamer 407 (temperature-sensitive), Gellan gum (ion-sensitive), and carbopol 934P (pH-sensitive). Other polymers, like sodium alginate and chitosan, offer benefits such as biocompatibility and antibacterial activity. Since these polymers have characteristics unique to themselves that impact formulation performance, they must be chosen or combined carefully to obtain the optimum formulation. Simultaneous delivery of an antimicrobial agent (e.g., metronidazole, doxycycline) with an anti-inflammatory agent (e.g., diclofenac sodium) has a dual benefit of reducing microbiologic burden and inflammation. In line with the 2017 AAP/EFP classification emphasizing individualization of treatment,in situ gels favor customization according to factors such as disease severity, risk of progression, and patient-specific issues. However, there are formulation challenges of low mechanical strength, low drug loading, and inconsistency of gelation. Research and development in polymer science on mucoadhesive agents, nanoparticles, and dual-responsive systems are already addressing these challenges.

 Therefore, in situ gels are intelligent and low-intervention methods for periodontal drug delivery. With increased research and clinical validation, they can emerge as a standard addition to the armamentarium of contemporary periodontics.

## Conclusion

 In situ gel-based drug delivery systems are revolutionizing the treatment of periodontal disease. They allow for localized, sustained, and controlled release of medication right in the periodontal pocket. Unlike traditional treatments, they deliver antibiotics and NSAIDs precisely where needed while keeping widespread exposure to a minimum. The mix of biocompatible polymers, innovative drug carriers, and advanced gelation methods opens up exciting possibilities for managing periodontitis effectively. However, we still need more clinical trials and advancements in formulation to make the most of this technology and get it into wider use. By incorporating responsive polymers and user-friendly designs, in situ gel technique could change periodontal therapy, providing a minimally invasive, cost-effective, and highly efficient treatment for millions affected by periodontal disease.

## Competing Interests

 The authors declare no personal, professional, or financial conflicts of interest that might influence the content of this paper.

## Data Availability

 No new data were created or analyzed in this study. Data sharing does not apply to this article as it is based solely on reviewing and analyzing previously published literature.

## Ethical Approval

 Since this review article does not involve human subjects, animal research, or clinical trials, ethical approval and informed consent are not needed. All information in this work arises from previously published research that has been properly referenced and credited.
